# Sparse observations induce large biases in estimates of the global ocean CO_2_ sink: an ocean model subsampling experiment

**DOI:** 10.1098/rsta.2022.0063

**Published:** 2023-06-26

**Authors:** Judith Hauck, Cara Nissen, Peter Landschützer, Christian Rödenbeck, Seth Bushinsky, Are Olsen

**Affiliations:** ^1^ Alfred-Wegener-Institut Helmholtz-Zentrum für Polar- und Meeresforschung, Bremerhaven, Germany; ^2^ Department of Atmospheric and Oceanic Sciences and Institute of Arctic and Alpine Research, University of Colorado Boulder, Boulder, CO, USA; ^3^ Flanders Marine Institute (VLIZ), Ostend, Belgium; ^4^ Max Planck Institut für Biogeochemie, Jena, Germany; ^5^ School of Ocean and Earth Science and Technology, University of Hawai’i at Mānoa, Department of Oceanography, Honolulu, HI, USA; ^6^ Geophysical Institute, University of Bergen, Bergen, Norway; ^7^ Bjerknes Centre for Climate Research, Bergen, Norway

**Keywords:** carbon dioxide, ocean carbon sink, pCO_2_ observations, observation system design

## Abstract

Estimates of ocean ${\mathrm{CO}}_{2}$ uptake from global ocean biogeochemistry models and ${p\mathrm{CO}}_{2}$-based data products differ substantially, especially in high latitudes and in the trend of the ${\mathrm{CO}}_{2}$ uptake since 2000. Here, we assess the effect of data sparsity on two ${p\mathrm{CO}}_{2}$-based estimates by subsampling output from a global ocean biogeochemistry model. The estimates of the ocean ${\mathrm{CO}}_{2}$ uptake are improved from a sampling scheme that mimics present-day sampling to an ideal sampling scheme with 1000 evenly distributed sites. In particular, insufficient sampling has given rise to strong biases in the trend of the ocean carbon sink in the ${p\mathrm{CO}}_{2}$ products. The overestimation of the ${\mathrm{CO}}_{2}$ flux trend by 20–35% globally and 50–130% in the Southern Ocean with the present-day sampling is reduced to less than 15% with the ideal sampling scheme. A substantial overestimation of the decadal variability of the Southern Ocean carbon sink occurs in one product and appears related to a skewed data distribution in ${p\mathrm{CO}}_{2}$ space. With the ideal sampling, the bias in the mean ${\mathrm{CO}}_{2}$ flux is reduced from 9–12% to 2–9% globally and from 14–26% to 5–17% in the Southern Ocean. On top of that, discrepancies of about $0.4\;{\mathrm{PgC}\;\mathrm{yr}}^{-1}$ (15%) persist due to uncertainties in the gas-exchange calculation.

This article is part of a discussion meeting issue ‘Heat and carbon uptake in the Southern Ocean: the state of the art and future priorities’.

## 1. Introduction

The ocean sequesters a remarkably constant fraction of about 25% of anthropogenic ${\mathrm{CO}}_{2}$ emissions each year [1,2]. This is quantified in the annual releases of the Global Carbon Budget (GCB) [2] through simulations with global ocean biogeochemistry models (GOBMs) and data products based on measurements of surface ocean ${\mathrm{pCO}}_{2}$ (${\mathrm{pCO}}_{2}$-products). This fraction is consistent with interior ocean biogeochemical observation-based estimates [3], atmospheric inversions [2] and observations of atmospheric $O_{2}$ to $N_{2}$ ratios [4]. There is agreement between the data classes on a mean ocean carbon uptake of 2–$2.2\;{\mathrm{PgC}\;\mathrm{yr}}^{-1}$ in the 1990s, on a stagnation of the ocean sink in the 1990s, and a reinvigoration since the early 2000s. However, there are discrepancies in the temporal evolution of the ocean sink, in particular between the GOBMs and ${p\mathrm{CO}}_{2}$ products [1,2]. The rate of increase 2000–2018 amounts to $0.41\pm 0.04\;{\mathrm{PgC}\;\mathrm{yr}}^{-1}\;{\mathrm{decade}}^{-1}$ in the GOBMs and to $0.69\pm 0.14\;{\mathrm{PgC}\;\mathrm{yr}}^{-1}\;{\mathrm{decade}}^{-1}$ in the ${p\mathrm{CO}}_{2}$ products (table 1). The discrepancy grows further in time, and is a factor of three for the trend since 2010 [1,2]: The ocean sink increased by $0.9\;{\mathrm{PgC}\;\mathrm{yr}}^{-1}\;{\mathrm{decade}}^{-1}$ since 2010 according to the ${p\mathrm{CO}}_{2}$ products, but only by $0.3\;{\mathrm{PgC}\;\mathrm{yr}}^{-1}\;{\mathrm{decade}}^{-1}$ according to the GOBMs [2]. The diverging trends since around 2000 stem from the Southern Ocean and the northern high latitudes [2]. The Southern Ocean also stands out as the region of largest discrepancy in the mean flux [1,2]. This discrepancy, however, lies within the uncertainty of the river flux adjustment [5] and its spatial distribution [6,7] that needs to be accounted for when comparing the ${\mathrm{pCO}}_{2}$ products’ flux estimate, which includes signals of carbon transport from land to ocean via rivers and associated ocean outgassing, with the GOBMs ocean sink estimate without this signal [1,2].

**Table 1.** Decadal trend in the ocean carbon uptake over the period 2000–2018 in the Global Carbon Budget 2022 [2] global ocean biogeochemistry models and surface ocean ${\mathrm{pCO}}_{2}$ based data products, and in the FESOM-1.4-REcoM2 version used here. The ensemble mean and standard deviation (s.d.) are also given.

dataset	trend 2000–2018 (${\mathrm{PgC}\;\mathrm{yr}}^{-1}\;{\mathrm{decade}}^{-1}$)
**global ocean biogeochemistry models used in Global Carbon Budget 2022**	
CESM2	0.40
NEMO3.6-PISCESv2-gas (CNRM)	0.44
FESOM2.1-REcoM2	0.45
NEMO-PISCES (IPSL)	0.38
MOM6-COBALT (Princeton)	0.37
MRI-ESM2-1	0.40
MICOM-HAMOCC (NorESM1-OCv1.2)	0.38
NEMO-PlankTOM12	0.50
CESM-ETHZ	0.40
MPIOM-HAMOCC6	0.34
ensemble mean±1 std	0.41±0.04
${\;}^{a}$FESOM-1.4-REcoM2	0.43
**${\text{pCO}}_{\text{2}}$ products**	
CMEMS-LSCE-FFNN	0.62
JMA-MLR	0.52
LDEO HPD	0.63
${\;}^{a}$MPI-SOM-FFN	0.95
NIES-NN	0.85
OS-ETHZ-GRaCER	0.60
${\;}^{a}$Jena CarboScope	0.67
ensemble mean±1 std	0.69±0.14

${\;}^{a}$The model and mapping methods used for this subsampling study.

Global ocean biogeochemistry models are general ocean circulation models with biogeochemical modules. They simulate the ${\mathrm{CO}}_{2}$ flux at the air–sea interface and the exchange of carbon between the surface and the deep ocean, with all their seasonal, interannual and longer time-scale variations. They further include a low- to intermediately complex representation of the biological carbon cycle [8]. They are closely tied to recent climate change and variability as they are forced with atmospheric reanalysis fields, such as the Japanese 55-year atmospheric reanalysis (JRA55-do) [9]. They reproduce the spatial and temporal variability of surface ocean ${p\mathrm{CO}}_{2}$ observations relatively well on large spatial and annual scales [1]. A direct comparison with ocean interior carbon accumulation 1994–2007 [3], as well as with the best estimate of the mean 1990s ocean carbon uptake as assessed by the Intergovernmental Panel on Climate Change (IPCC) fourth Assessment Report (AR4) [10] indicates that the GOBMs underestimate the mean ocean carbon sink by 0.2–$0.4\;{\mathrm{PgC}\;\mathrm{yr}}^{-1}$ [2]. A similar underestimation of ocean carbon uptake in Earth System Models was related to model biases in surface to deep ocean carbon transport [11–13] and in the surface chemical buffer capacity (Revelle factor) [14,15].

Annually updated estimates of the ocean carbon sink from surface ocean ${p\mathrm{CO}}_{2}$ observations have been facilitated by the advent of the quality-controlled Surface Ocean ${\mathrm{CO}}_{2}$ Atlas (SOCAT) in 2011 [16,17] and its annual updates since 2015 [18]. The SOCATv2022 release contains 33.7 million ${p\mathrm{CO}}_{2}$ observations with an estimated accuracy of better than 5 micro atmospheres. These observations are, however, unevenly distributed in space, time and seasons ([18], updated). They cover about 2% of all grid points in an array with monthly $1^{\circ }\;\mathrm{longitude}\times 1^{\circ }$ latitude dimensions, 1990 to present. Based on this dataset, mapping methods were developed that can fill the gaps (98%) to obtain full global coverage. The ocean ${\mathrm{CO}}_{2}$ uptake can be calculated from these global maps of surface ocean ${\mathrm{pCO}}_{2}$ with gas-exchange parametrizations [19,20], observation-derived wind speed data (e.g. from atmospheric reanalysis) and sea surface temperature and salinity. The first methods, based on a data-driven mixed-layer scheme, a neural network, and multi-linear regression were published in 2013/2014 [21–24] and others have followed since then [25–28]. While a first comparison of the different methods identified substantial discrepancies in terms of the amplitude of interannual variations [29], evaluation with independent data indicated that data scarcity presents a larger limitation than potential methodological weaknesses [26]. This is supported by model subsampling experiments that have revealed biases in ${p\mathrm{CO}}_{2}$ products due to data scarcity [30–33]. Specifically, insufficient sampling leads to an overestimation of the global and Southern Ocean amplitude of decadal variability by 21% and 31%, respectively [31], and to a 30% overestimation of the mean flux in the Atlantic Ocean [32]. These studies, however, could not assess the confidence in the ocean carbon sink trend estimate since 2000. This is because one study was based on a large-ensemble Earth System Model testbed of a high emission future scenario [31], which is not comparable to observed atmospheric ${\mathrm{CO}}_{2}$ trends in the period 2000–2018, while the other studies were confined to single ocean basins [32,33].

GOBMs were used for many years as the only data class for the ocean carbon sink estimate in the GCB. Ocean carbon sink estimates from ${p\mathrm{CO}}_{2}$ products were used as an independent comparison dataset for the GOBMs since 2013 [34]. In the budget 2021, the ocean sink estimate was for the first time obtained as the average of the GOBM ensemble mean (eight GOBMs) and the ${p\mathrm{CO}}_{2}$-product ensemble mean (seven ${p\mathrm{CO}}_{2}$ products) [2]. Sparsity in surface ${p\mathrm{CO}}_{2}$ observations is thus one of the major sources of uncertainties for this combined ocean sink estimate, and particularly for the temporal evolution, including the trend since around 2000. Autonomous observations, e.g. from biogeochemical Argo floats (bgcArgo) [35,36] or uncrewed surface vehicles [37] may potentially close gaps in the observational network [32,33]. This is of particular interest in southern high latitudes that are less regularly accessed by ships, in particular in winter, although the lower accuracy of ${p\mathrm{CO}}_{2}$ values derived from float-based pH measurements remains a major challenge [30,35,38].

In this study, we use output from one global ocean biogeochemistry model that contributes to the Global Carbon Budget to assess the effect of data sparsity on the ${p\mathrm{CO}}_{2}$-based estimates of the ocean carbon sink. We train two mapping methods with ${p\mathrm{CO}}_{2}$ and other environmental data from the GOBM, which was subsampled according to three sampling schemes (SOCAT, SOCAT plus Southern Ocean bgcArgo floats, ideal bgcArgo coverage). Comparing the resulting ${p\mathrm{CO}}_{2}$ reconstructions and air–sea ${\mathrm{CO}}_{2}$ fluxes to the known model truth, we assess reconstruction biases with respect to the mean flux, its variability and the magnitude of its trend 2000–2018.

## 2. Methods

### (a) Ocean biogeochemistry model simulation

We use the ocean circulation model FESOM1.4 [39] coupled to the ocean biogeochemistry model REcoM2 [1,40,41]. The unstructured mesh has 126 859 surface nodes, roughly equivalent to a $1^{\circ }\times 1^{\circ }$ resolution. The surface nodes are unevenly distributed with the lowest resolution in the subtropics, and higher resolution at the equator, the coasts, the southern high and in particular, the northern high latitudes [39]. The model is started from initial conditions (World Ocean Atlas for nutrient fields [42], GLODAPv2 for alkalinity and preindustrial dissolved inorganic carbon [43]). It is spun up from 1850 to 1957 using repeated year atmospheric forcing, taken from the year 1961. The atmospheric forcing fields for the spin-up and for the simulation period 1958–2018 are taken from the JRA55-do Reanalysis Version 1.4.0 [9]. Further, both spin-up and simulation periods are forced with observed atmospheric ${\mathrm{xCO}}_{2}$ as provided by the GCB [44], which is the average of atmospheric ${\mathrm{CO}}_{2}$ measurements from the Mauna Loa and South Pole stations since 1958 [45,46]. This is converted to ${p\mathrm{CO}}_{2}$ using spatiotemporally varying sea-level pressure and the water vapour correction (a function of sea surface temperature and salinity). Carbonate chemistry and air–sea ${\mathrm{CO}}_{2}$ exchange are calculated with the mocsy routines [47] that apply a quadratic gas-exchange parameterization [20] (see equations (2.1) and (2.2)). This is the same model version as used in the Global Carbon Budget 2020 [44] and the RECCAP project (https://reccap2-ocean.github.io). The model output was interpolated to a $1^{\circ }\times 1^{\circ }$ field using bilinear interpolation.

### (b) Sampling masks

**Figure 1.**
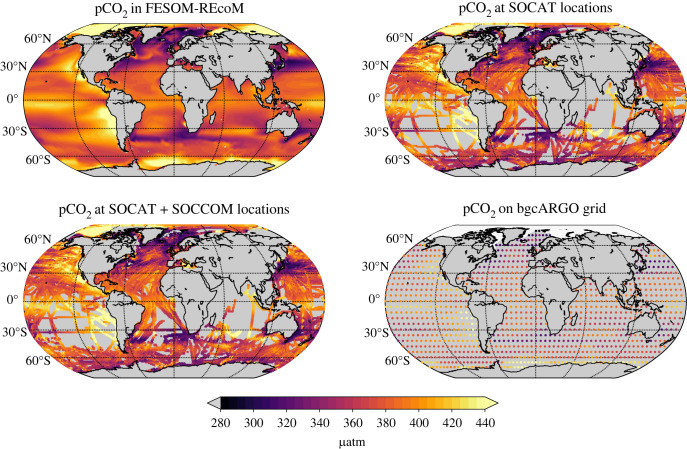
Annual mean partial pressure of ${\mathrm{CO}}_{2}$ (${p\mathrm{CO}}_{2}$) averaged over the period 2009–2018 from the output from the global ocean biogeochemistry model FESOM-REcoM. Upper left figure shows the full model output, and the other three panels show the model output after subsampling according to the three masks, as indicated in the title (SOCAT, SOCAT+SOCCOM, bgcArgo). In the bgcArgo panel, the white background shading in the Arctic depicts the Arctic region that is excluded from all analyses. See text for more explanation.

**Figure 2.**
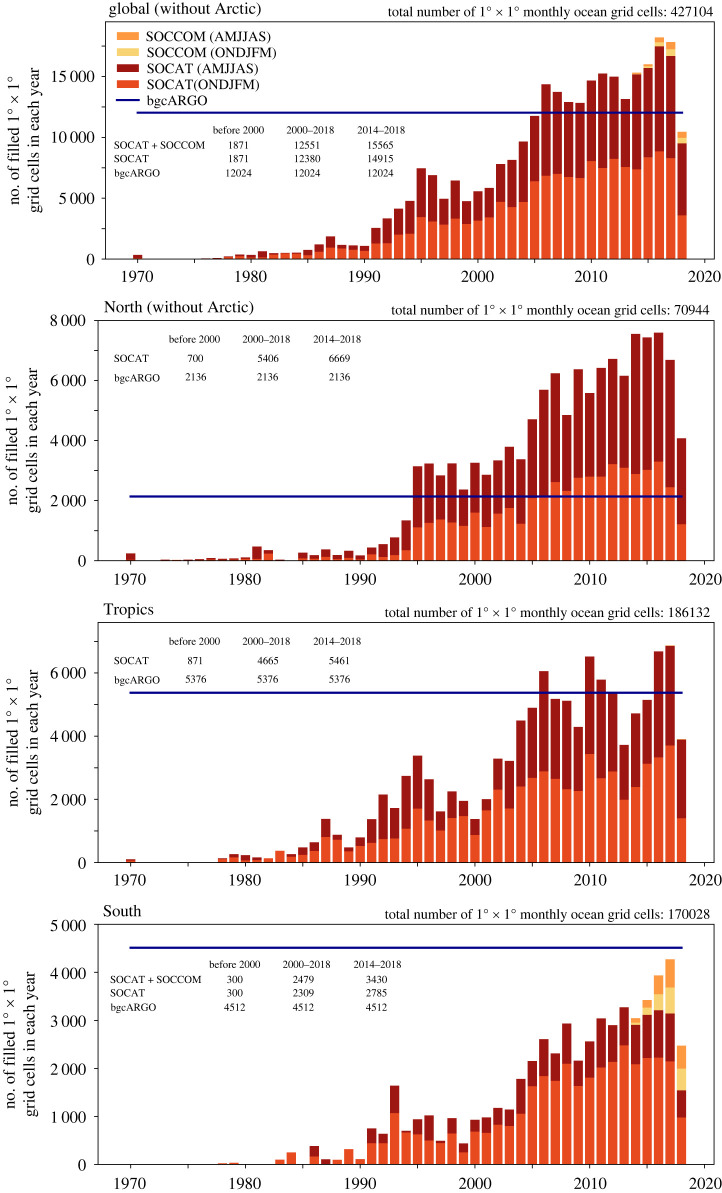
Number of monthly $1^{\circ }\times 1^{\circ }$ grid cells covered per year. Shown is the data coverage in the global ocean (top) and three large-scale regions, top to bottom: North (north of $30^{\circ }N$, excluding the Arctic), Tropics ($30^{\circ }S$ to $30^{\circ }N$), South (south of $30^{\circ }S$). The bars illustrate the data coverage from the SOCAT (dark orange: October to March (Southern Hemisphere summer, Northern Hemisphere winter), dark red: April to September) and SOCCOM (yellow: October to March light orange: April to September) data sets. The hypothetical ideal bgcArgo data coverage is constant in time as indicated by the dark blue horizontal line. Note the different axis scales. Average number of filled grid cells is given within the figures for the periods before 2000, 2000–2018 and 2014–2018. Total number of monthly $1^{\circ }\times 1^{\circ }$ ocean grid cells per region is given at the top of the panels.

We create three sampling masks (figure 1). The first mask is based on SOCATv2019 (gridded version) [18] and thus comparable to the current ${p\mathrm{CO}}_{2}$ product submissions to the Global Carbon Budget. A second mask is created from SOCATv2019 and the bgcArgo observations of the Southern Ocean Carbon and Climate Observations and Modeling project (SOCCOM). The SOCCOM floats provide summer and winter data since 2014. Over the entire study period, this mask is dominated by SOCAT, and SOCCOM contributes less than a quarter of Southern Ocean observation since 2014 (figure 2). Finally, the third mask is based on the ideal bgcArgo sampling scheme with 1000 bgcArgo floats which corresponds to a $6^{\circ }\times 6^{\circ }$ regular grid [36]. Notably, even though the ideal bgcArgo design aims for full global coverage, the floats do not extend into the Arctic realm following Roemmich *et al.* [36]. We therefore chose to not place hypothetical floats there, using the RECCAP Arctic mask (https://reccap2-ocean.github.io/), which is depicted by lighter background shading in figure 1*d*. In the following, we refer to this ideal sampling grid as bgcArgo. We compare the actual temporally varying data availability from SOCAT and SOCCOM to a hypothetical case with full ideal bgcArgo sampling since 1970. We do not account for movement of floats in the bgcArgo case. We also do not account for higher uncertainty of bgcArgo derived ${p\mathrm{CO}}_{2}$ values.

The mean ${p\mathrm{CO}}_{2}$ over the period 2009–2018 for the original model output and the three sampling schemes is illustrated in figure 1. The difference in annual mean ${p\mathrm{CO}}_{2}$ in the Southern Ocean between full model output and ${p\mathrm{CO}}_{2}$ at SOCAT (and SOCAT+SOCCOM) locations can be explained by the summer bias in SOCAT (see electronic supplementary material, figures S1 and S2). The data coverage increases globally from SOCAT to SOCAT+SOCCOM to bgcArgo, except for the Arctic (by design). We therefore exclude the Arctic from our analysis, again using the RECCAP Arctic mask. The number of observations from the three data sources (SOCAT, SOCCOM, bgcArgo) is presented in figure 2. The ideal (hypothetical) bgcArgo grid provides more filled grid cells than SOCAT until 2004 in the global case, until 1994 for the North (north of $30^{\circ }N$, Arctic excluded), until 2005 in the tropics (and intermittently thereafter, $30^{\circ }S$ to $30^{\circ }N$), and always in the Southern Ocean (south of $30^{\circ }S$). This picture holds throughout seasons, except in the Southern Ocean. Here, SOCAT can offer more summer grid cells covered than bgcArgo since 2006, but only about half the amount of grid cell coverage in winter (electronic supplementary material, Figures S3 and S4). Globally, coverage corresponds to 2.4% of all monthly $1^{\circ }\times 1^{\circ }$ grid cells in the bgcArgo scheme, whereas SOCAT would cover 2.6% in the period 2000–2018 and 3.1% in the last five years 2014-2018 (3.3% SOCAT+SOCCOM). The majority of observations come from the North, where 7.6% of the spatio-temporal field 2000–2018 was covered with SOCAT (3.0% in the bgcArgo scheme). In the same period, SOCAT coverage amounts to 2.5% (2.9% bgcArgo) in the tropics, and to 1.4% (2.7% bgcArgo) in the Southern Ocean (figure 2).

**Figure 3.**
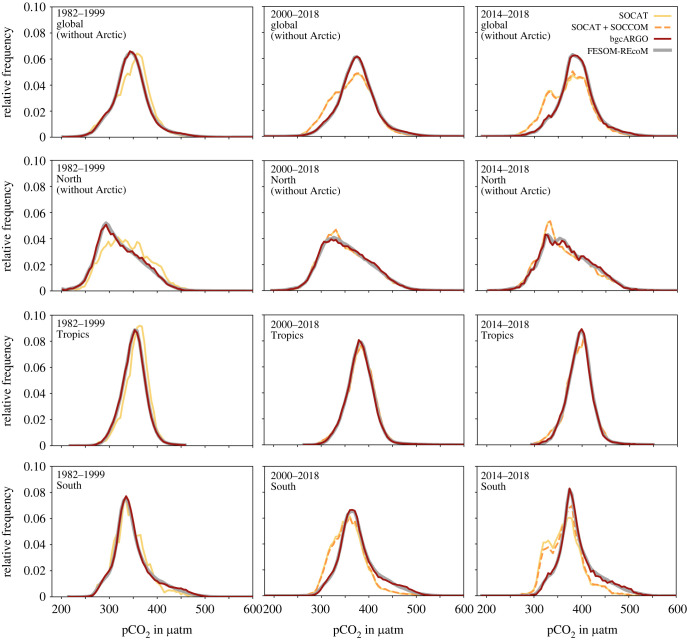
Data distribution in ${\mathrm{pCO}}_{2}$ space for the regions: (top to bottom) global (no Arctic), North (no Arctic), Tropics, and South, and for the periods (from left to right) 1982–2000, 2000–2018 and the last 5 years 2014–2018. The thick grey line depicts the full model output, and the coloured lines the three sampling masks (SOCAT: yellow; SOCAT+SOCCOM: dashed orange; bgcArgo: red).

The data distribution in ${\mathrm{pCO}}_{2}$ space also varies between the sampling schemes (figure 3). The SOCAT and SOCAT+SOCCOM sampling masks result in a skewed data distribution relative to the full FESOM-REcoM output. In particular, ${\mathrm{pCO}}_{2}$ is skewed towards higher ${\mathrm{pCO}}_{2}$ values before 2000 in all regions, and towards lower ${\mathrm{pCO}}_{2}$ values after 2000. The latter is most pronounced in the Southern Ocean and hence also in the global mean, but also has a contribution from the north. The bgcArgo scheme captures the data distribution well throughout all time periods and regions.

### (c) Mapping methods

We use two mapping methods that were among the first included in the GCB (CarboScope [48], MPI-SOM-FFN [49]). Both methods produce global maps of seawater ${\mathrm{pCO}}_{2}$ (${\mathrm{pCO}}_{2}^{sw}$), and the ${\mathrm{CO}}_{2}$ flux (${\mathrm{FCO}}_{2}$) is calculated from the gas transfer velocity $k_{w}$, the solubility of ${\mathrm{CO}}_{2}$ in seawater ($K_{0}$) and the difference between ${\mathrm{pCO}}_{2}$ in seawater and in the atmosphere (${\mathrm{pCO}}_{2}^{\mathrm{atm}}$) [20]:

2.1 $${\mathrm{FCO}}_{2}=k_{w}\cdot K_{0}\cdot ({\mathrm{pCO}}_{2}^{sw}-{\mathrm{pCO}}_{2}^{\mathrm{atm}}).$$

The gas transfer velocity is calculated as a function of wind speed (U), the coefficient of gas transfer (a), the temperature-dependent Schmidt number (Sc) and is scaled with the ice-free area ($1{-F}_{\mathrm{ice}}$):

2.2 $$k_{w}=a\cdot U^{2}\cdot (1-F_{\mathrm{ice}})\cdot {\frac{660}{\mathrm{Sc}}}^{0.5}.$$

The CarboScope ${\mathrm{pCO}}_{2}$ interpolation (also known as Jena mixed layer scheme, Jena-MLS) is a hybrid scheme that uses a multi-linear regression model to link ocean internal carbon sources and sinks to environmental variables, and an explicit interannual correction when ${\mathrm{pCO}}_{2}$ observations are available [48]. The following input fields for the regression and for the parametrizations of gas exchange and carbonate chemistry, normally taken from observation-based data sets, are replaced here by FESOM-REcoM output (interpolated from monthly to daily time-steps, and from $1^{\circ }\times 1^{\circ }$ to $2.5^{\circ }\;\mathrm{longitude}\times 2^{\circ }$ latitude): sea surface temperature, sea surface salinity, ice-fraction, alkalinity and mixed layer depth. Wind speed and sea-level pressure are taken from JRA55-do both in FESOM-REcoM and the standard CarboScope ${\mathrm{pCO}}_{2}$ interpolation. Usually, CarboScope uses an alkalinity climatology based on sea surface salinity and temperature [50]. In our application, the time-varying FESOM-REcoM alkalinity is used. Likewise, the ${\mathrm{pCO}}_{2}$ data are subsampled from the surface ${\mathrm{pCO}}_{2}$ field of FESOM-REcoM. In addition, CarboScope uses a prior estimate of the decadal trend of the ${\mathrm{CO}}_{2}$ flux taken from an ocean inverse model (OCIM, [51]). In an additional test case, the prior is replaced with the decadal trend of a FESOM-REcoM simulation forced by increasing atmospheric ${\mathrm{CO}}_{2}$ and repeated year atmospheric forcing fields (1961, referred to as simulation C in the Global Carbon Budget and RECCAP, no climate change and variability). The scheme thereby attempts to reconstruct the surface ${\mathrm{pCO}}_{2}$ fields 1958–2019.

We use two realizations of the CarboScope product. The first realization calculates the air–sea ${\mathrm{CO}}_{2}$ flux at the daily time-step used in CarboScope with the gas-transfer velocity from FESOM-REcoM, which was calculated at every 15 min time step and saved as monthly kw660, i.e. normalized to a Schmidt number of 660. The temperature dependence (varying Schmidt number) is taken into account based on the monthly FESOM-REcoM sea surface temperature output. ${\mathrm{CO}}_{2}$ solubility is also calculated from FESOM-REcoM sea surface temperature and salinity fields. These daily ${\mathrm{CO}}_{2}$ flux fields are then averaged into monthly fields. The gas-transfer velocity in FESOM-REcoM has a mean value of $14.0\;{\mathrm{cm}\;h}^{-1}$. Atmospheric ${\mathrm{pCO}}_{2}$ is also taken from FESOM-REcoM, i.e. from a globally uniform ${\mathrm{xCO}}_{2}$ value converted to ${\mathrm{pCO}}_{2}$ with spatiotemporally varying sea-level pressure, sea surface temperature and salinity. The second realization uses the native CarboScope gas-exchange formulation based on the quadratic wind speed dependence [52] with the transfer coefficient scaled to match a global mean transfer velocity of $16.5\;{\mathrm{cm}\;h}^{-1}$ [53] and the spatially resolved atmospheric ${\mathrm{CO}}_{2}$ fields from the CarboScope atmospheric inversion [48]. The same wind fields (JRA55-do reanalysis) as in FESOM-REcoM are used. In CarboScope, the surface ${\mathrm{pCO}}_{2}$ differs between the two realizations as the ocean internal sources/sinks may respond to the air–sea ${\mathrm{CO}}_{2}$ flux.

The MPI-SOM-FFN method uses a self-organizing map (SOM) approach to divide the global ocean into large-scale and highly dynamic biogeochemical provinces, in which ${\mathrm{pCO}}_{2}$ is reconstructed with a feed-forward neural network (FFN) model, that has previously been trained with ${\mathrm{pCO}}_{2}$ observations and environmental variables [49]. Here, the monthly $1^{\circ }\times 1^{\circ }$ MPI-SOM-FFN reconstruction 1982–2018 uses chlorophyll a, sea surface temperature, sea surface salinity, atmospheric ${\mathrm{CO}}_{2}$ concentration and mixed layer depth as environmental variables from FESOM-REcoM model output. By training the feed-forward neural network model on ${\mathrm{pCO}}_{2}$ data from the FESOM-REcoM model that is subsampled according to the experiment setup of this study, the network reconstructs a nonlinear relationship between environmental drivers and ${\mathrm{pCO}}_{2}$ within each biome, that is then used to fill gaps in the sea surface ${\mathrm{pCO}}_{2}$.

As for CarboScope, we use two air–sea ${\mathrm{CO}}_{2}$ flux datasets for MPI-SOM-FFN that both build on the same monthly $1^{\circ }\times 1^{\circ }$ ${\mathrm{pCO}}_{2}$ fields, and on FESOM-REcoM output of atmospheric ${\mathrm{pCO}}_{2}$. Note that MPI-SOM-FFN has no feedbacks between ${\mathrm{CO}}_{2}$ flux and ${\mathrm{pCO}}_{2}$, in contrast to CarboScope. Firstly, ${\mathrm{CO}}_{2}$ flux is calculated with FESOM-REcoM output (kw660, sea surface temperature, sea surface salinity) according to equations (2.1) and (2.2) and we thereby take into account the temperature dependency of the gas-transfer velocity as in the model equations [20,47]. Secondly, following the native MPI-SOM-FFN methodology, the air–sea ${\mathrm{CO}}_{2}$ exchange is calculated with a quadratic gas-exchange velocity parametrization [52], scaled to a global mean transfer velocity $\left\langle k_{w}\right\rangle$ of $16.5\;{\mathrm{cm}\;h}^{-1}$. The solubility of ${\mathrm{CO}}_{2}$ in seawater is calculated from sea surface temperature and salinity output from FESOM-REcoM and the formulation of Weiss [54]. The transfer velocity $k_{w}$ is calculated from ERA5 6-hourly u- and v-wind components from which the square of wind speed (i.e. $U^{2}$) is calculated at 6-hourly temporal resolution. The second moment wind speed is then obtained by averaging into monthly means ($\left\langle U^{2}\right\rangle$) before calculating gas-exchange according to equations (2.1) and (2.2). Additionally, the air–sea ${\mathrm{CO}}_{2}$ exchange is limited to ice-free ocean areas, determined by the sea-ice fraction provided by the FESOM-REcoM model output.

### (d) Statistics

We primarily compare the mean ${\mathrm{CO}}_{2}$ flux estimate over the last decade of the reconstructions (2009–2018, ${\mathrm{FCO}}_{2}^{2009\text{--}2018}$) and the trend 2000–2018 which is calculated as a linear fit over these years. We further calculate the standard deviation and correlation coefficient of the detrended ${\mathrm{CO}}_{2}$ flux time-series 1982–2018 as a measure of the amplitude and phasing of interannual variability of the large-scale fluxes [2,29], acknowledging that the so-calculated metrics also contain signals from decadal variability.

## 3. Results

The results section is structured into three parts. Firstly, we analyse the air–sea ${\mathrm{CO}}_{2}$ fluxes and the mismatch between ${\mathrm{pCO}}_{2}$ products and known model truth based on the air–sea ${\mathrm{CO}}_{2}$ fluxes calculated from mapped ${\mathrm{pCO}}_{2}$ and the FESOM-REcoM gas-exchange formulation (§3a). This is to analyse the effect of sampling distribution without interference of gas-exchange calculations. Secondly, we compare differences in reconstructed surface ocean ${\mathrm{pCO}}_{2}$ (§3b). Finally, we analyse the difference between using the ocean model’s or the ${\mathrm{pCO}}_{2}$ products’ native gas-exchange formulation for the calculation of air–sea ${\mathrm{CO}}_{2}$ flux from mapped ${\mathrm{pCO}}_{2}$ (§3c).

### (a) Air–sea ${\mathrm{CO}}_{2}$ fluxes

**Figure 4.**
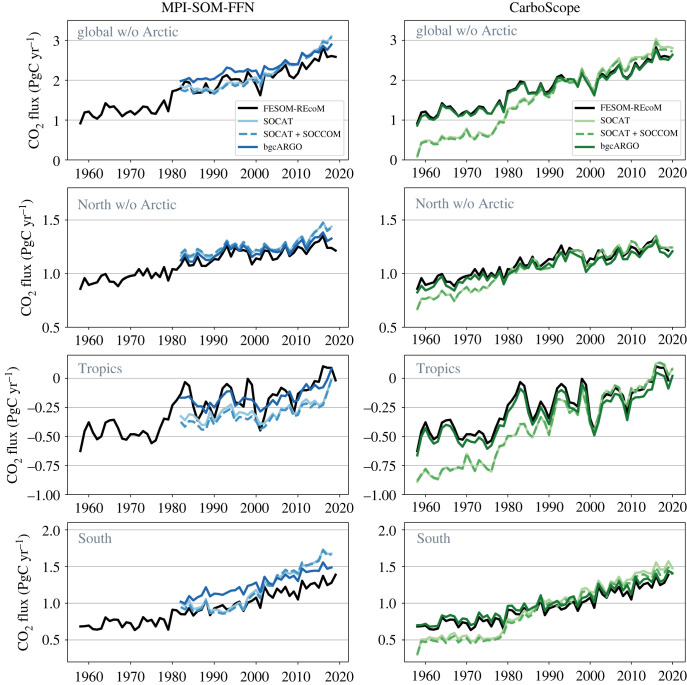
Annual mean air-to-sea ${\mathrm{CO}}_{2}$ flux (${\mathrm{PgC}\;\mathrm{yr}}^{-1}$) from the ${\mathrm{pCO}}_{2}$ reconstructions (coloured lines) compared with the original FESOM-REcoM ${\mathrm{CO}}_{2}$ flux (black line). The left column shows the MPI-SOM-FFN and the right column the CarboScope reconstructions for the three sampling schemes as indicated in the figures. MPI-SOM-FFN and CarboScope fluxes are calculated from mapped ${\mathrm{pCO}}_{2}$ and FESOM-REcoM gas-transfer velocity. From top to bottom: Global without Arctic, North (north of $30^{\circ }N$) with Arctic excluded, Tropics ($30^{\circ }S$ to $30^{\circ }N$), South (south of $30^{\circ }S$). Positive fluxes denote a flux into the ocean.

**Figure 5.**
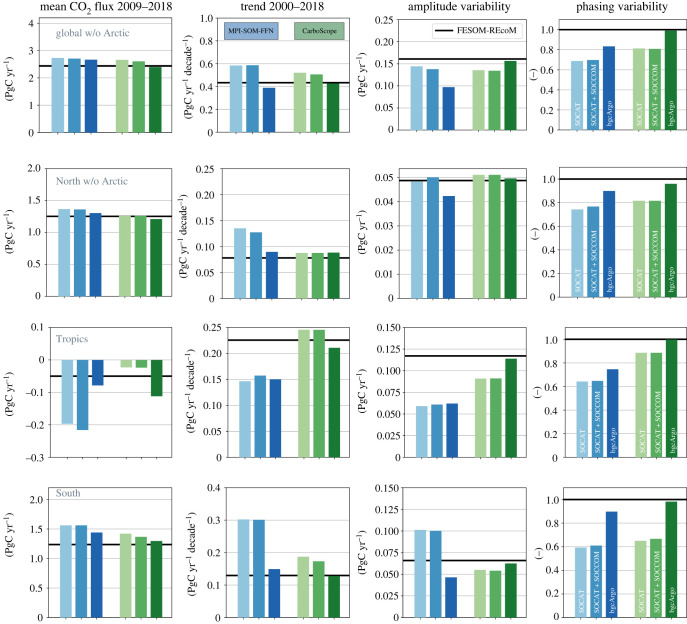
Effect of sampling distribution on statistics of annual mean air-to-sea ${\mathrm{CO}}_{2}$ flux (${\mathrm{PgC}\;\mathrm{yr}}^{-1}$) calculated from the ${\mathrm{pCO}}_{2}$ reconstructions with FESOM-REcoM gas-transfer velocity (coloured bars) compared with the original FESOM-REcoM ${\mathrm{CO}}_{2}$ flux (black line). From left to right: Mean ${\mathrm{CO}}_{2}$ flux 2009–2018 (${\mathrm{PgC}\;\mathrm{yr}}^{-1}$), trend 2000–2018 (${\mathrm{PgC}\;\mathrm{yr}}^{-1}\;{\mathrm{decade}}^{-1}$), amplitude of variability (standard deviation of detrended time-series) 1982–2018, phasing of variability (correlation coefficient of detrended time-series) 1982–2018. The blue bars show the MPI-SOM-FFN and the green bars the CarboScope reconstructions for the three sampling schemes as indicated in the figures (SOCAT: light colour, SOCAT+SOCCOM: medium colour, bgcArgo: dark colour). These statistics are calculated from the area-integrated time-series for (top to bottom): global excluding the Arctic, North excluding the Arctic, Tropics, South.

The reconstructed global and regional ${\mathrm{CO}}_{2}$ flux time-series from the three sampling experiments with the same gas-exchange calculation as in FESOM-REcoM are presented in figure 4, and the key statistics of the global ${\mathrm{CO}}_{2}$ flux in figure 5. Both mapping methods overestimate the mean ${\mathrm{CO}}_{2}$ uptake 2009–2018 and the trend 2000–2018 in the SOCAT sampling scheme. In the MPI-SOM-FFN method, the 12% overestimation of the mean in the SOCAT scheme is reduced to 9% in bgcArgo. The 9% overestimation in CarboScope (SOCAT) vanishes in the bgcArgo scheme (2% underestimation, figures 4 and 5). For the trend since 2000, the overestimation of 35% in the SOCAT scheme turns into a 10% underestimation with the bgcArgo scheme for MPI-SOM-FFN and the 20% overestimation to a 2% underestimation in CarboScope. Increasing data availability from SOCAT to the bgcArgo scheme hence leads to substantially improved agreement on the trend since 2000 (FESOM-REcoM: $0.43\;{\mathrm{PgC}\;\mathrm{yr}}^{-1}\;{\mathrm{decade}}^{-1}$, MPI-SOM-FFN (SOCAT): $0.58\;{\mathrm{PgC}\;\mathrm{yr}}^{-1}\;{\mathrm{decade}}^{-1}$, (bgcArgo): $0.39\;{\mathrm{PgC}\;\mathrm{yr}}^{-1}\;{\mathrm{decade}}^{-1}$, CarboScope (SOCAT): $0.52\;{\mathrm{PgC}\;\mathrm{yr}}^{-1}\;{\mathrm{decade}}^{-1}$, (bgcArgo): $0.43\;{\mathrm{PgC}\;\mathrm{yr}}^{-1}\;{\mathrm{decade}}^{-1}$), and on the mean flux 2009–2018 in both products (FESOM-REcoM: $2.44\;{\mathrm{PgC}\;\mathrm{yr}}^{-1}$, MPI-SOM-FFN (SOCAT): $2.73\;{\mathrm{PgC}\;\mathrm{yr}}^{-1}$, (bgcArgo): $2.67\;{\mathrm{PgC}\;\mathrm{yr}}^{-1}$, CarboScope (SOCAT): $2.66\;{\mathrm{PgC}\;\mathrm{yr}}^{-1}$, (bgcArgo): $2.39\;{\mathrm{PgC}\;\mathrm{yr}}^{-1}$). The difference between the reconstructions based on SOCAT and SOCAT+SOCCOM sampling schemes is small (global and Southern Ocean ${\mathrm{FCO}}_{2}^{2009\text{--}2018}\;0.04\text{--}0.05\;{\mathrm{PgC}\;\mathrm{yr}}^{-1}$). The amplitude of variability is with $0.14\;{\mathrm{PgC}\;\mathrm{yr}}^{-1}$ relatively well captured by MPI-SOM-FFN (SOCAT) in comparison to FESOM-REcoM ($0.16\;{\mathrm{PgC}\;\mathrm{yr}}^{-1}$) and is somewhat underestimated in the bgcArgo sampling scheme ($0.10\;{\mathrm{PgC}\;\mathrm{yr}}^{-1}$). The amplitude of variability of CarboScope turns from a slight underestimation with SOCAT sampling ($0.14\;{\mathrm{PgC}\;\mathrm{yr}}^{-1}$) to a good match with bgcArgo sampling ($0.16\;{\mathrm{PgC}\;\mathrm{yr}}^{-1}$). The CarboScope method reproduces the phasing of temporal variability, as measured by the correlation coefficient with the FESOM-REcoM annual time-series, more skilfully than MPI-SOM-FFN, and responds more strongly to higher and more evenly distributed data availability (MPI-SOM-FFN (SOCAT): 0.69, (bgcArgo): 0.83, CarboScope (SOCAT): 0.81, (bgcArgo): 0.99).

A notable result is found in the Southern Ocean (figure 4). Here, the overestimation of the trend with the SOCAT sampling scheme of 50% in CarboScope and 130% in MPI-SOM-FFN is eliminated with the bgcArgo scheme (trend 2000–2018 FESOM-REcoM: $0.13\;{\mathrm{PgC}\;\mathrm{yr}}^{-1}\;{\mathrm{decade}}^{-1}$, MPI-SOM-FFN SOCAT: $0.30\;{\mathrm{PgC}\;\mathrm{yr}}^{-1}\;{\mathrm{decade}}^{-1}$, bgcArgo: $0.15\;{\mathrm{PgC}\;\mathrm{yr}}^{-1}\;{\mathrm{decade}}^{-1}$; CarboScope (SOCAT): $0.19\;{\mathrm{PgC}\;\mathrm{yr}}^{-1}\;{\mathrm{decade}}^{-1}$, bgcArgo: $0.13\;{\mathrm{PgC}\;\mathrm{yr}}^{-1}\;{\mathrm{decade}}^{-1}$). Similarly, the amplitude of variability is reduced by a factor of two in MPI-SOM-FFN in the bgcArgo relative to SOCAT sampling scheme. The correlation with the FESOM-REcoM time-series increases in both products with the bgcArgo scheme (MPI-SOM-FFN: from 0.59 to 0.90, CarboScope: from 0.65 to 0.98). Interestingly, an erroneous acceleration of the Southern Ocean carbon sink since the late 1990s in MPI-SOM-FFN is corrected by increased data availability. The mean flux in the bgcArgo scheme is then, however, consistently overestimated by about $0.2\;{\mathrm{PgC}\;\mathrm{yr}}^{-1}$ (16%, 2009–2018) in MPI-SOM-FFN. The temporal evolution of CarboScope after 1980 is not as strongly affected as in MPI-SOM-FFN, although the mean ${\mathrm{FCO}}_{2}^{2009\text{--}2018}$ and particularly the trend are also reduced in CarboScope. The bgcArgo sampling scheme corrects a low bias in the CarboScope ${\mathrm{CO}}_{2}$ flux prior to 1985.

An improvement of the reconstructions with increased and more evenly distributed data availability also occurs in the tropics (figure 4). In the bgcArgo scheme, the mean ${\mathrm{FCO}}_{2}^{2009\text{--}2018}$ is well captured with a slight overestimation of the ocean carbon outgassing by both products (FESOM-REcoM: $-0.05\;{\mathrm{PgC}\;\mathrm{yr}}^{-1}$, MPI-SOM-FFN: $-0.08\;{\mathrm{PgC}\;\mathrm{yr}}^{-1}$, CarboScope: $-0.11\;{\mathrm{PgC}\;\mathrm{yr}}^{-1}$). This is a substantial improvement compared with the SOCAT scheme in MPI-SOM-FFN ($-0.20\;{\mathrm{PgC}\;\mathrm{yr}}^{-1}$), whereas the offset is somewhat larger and of opposite sign for CarboScope in the bgcArgo sampling scheme (SOCAT, $-0.02\;{\mathrm{PgC}\;\mathrm{yr}}^{-1}$). The phasing of temporal variability improves in the bgcArgo scheme (correlation coefficients MPI-SOM-FFN: from 0.64 to 0.75, CarboScope: from 0.89 to 1.00). The amplitude of variability is too low in both products, but the discrepancy is eliminated in CarboScope with the bgcArgo scheme. The availability of data prior to 1990 in the bgcArgo scheme corrects a large tropical outgassing signal in CarboScope based on SOCAT (and SOCCOM, figure 4), which we relate to the very low data availability before 1990 and to the skewed SOCAT ${\mathrm{pCO}}_{2}$ distribution towards higher ${\mathrm{pCO}}_{2}$ (figure 3, tropics, 1982–1999).

In the North (north of $30^{\circ }N$, Arctic excluded), there is little improvement from SOCAT to ideal bgcArgo sampling for the mean ${\mathrm{CO}}_{2}$ uptake and amplitude of variability (figures 4 and 5). This is because more observed grid cells have been available in SOCAT since 1995 than in bgcArgo (figure 2). The reconstructed mean flux in MPI-SOM-FFN bgcArgo scheme is 4% higher than in the FESOM-REcoM data. Interestingly, the effect of the skewed SOCAT distribution (figure 3) on the ${\mathrm{CO}}_{2}$ flux in the North is small, but notable for the last 5 years and the trend in MPI-SOM-FFN. The overestimation of the trend (2000–2018) is reduced in MPI-SOM-FFN with the ideal sampling (FESOM-REcoM: $0.08\;{\mathrm{PgC}\;\mathrm{yr}}^{-1}\;{\mathrm{decade}}^{-1}$, MPI-SOM-FFN (SOCAT): $0.14\;{\mathrm{PgC}\;\mathrm{yr}}^{-1}\;{\mathrm{decade}}^{-1}$, MPI-SOM-FFN (bgcArgo): $0.09\;{\mathrm{PgC}\;\mathrm{yr}}^{-1}\;{\mathrm{decade}}^{-1}$; no change in CarboScope: $0.09\;{\mathrm{PgC}\;\mathrm{yr}}^{-1}\;{\mathrm{decade}}^{-1}$) and the correlation is increased for MPI-SOM-FFN and CarboScope (MPI-SOM-FFN: from 0.74 to 0.90, CarboScope: from 0.82 to 0.96). As in the other regions, an underestimation of the CarboScope ${\mathrm{CO}}_{2}$ flux before 1980 in the SOCAT scheme is corrected with the bgcArgo scheme.

**Figure 6.**
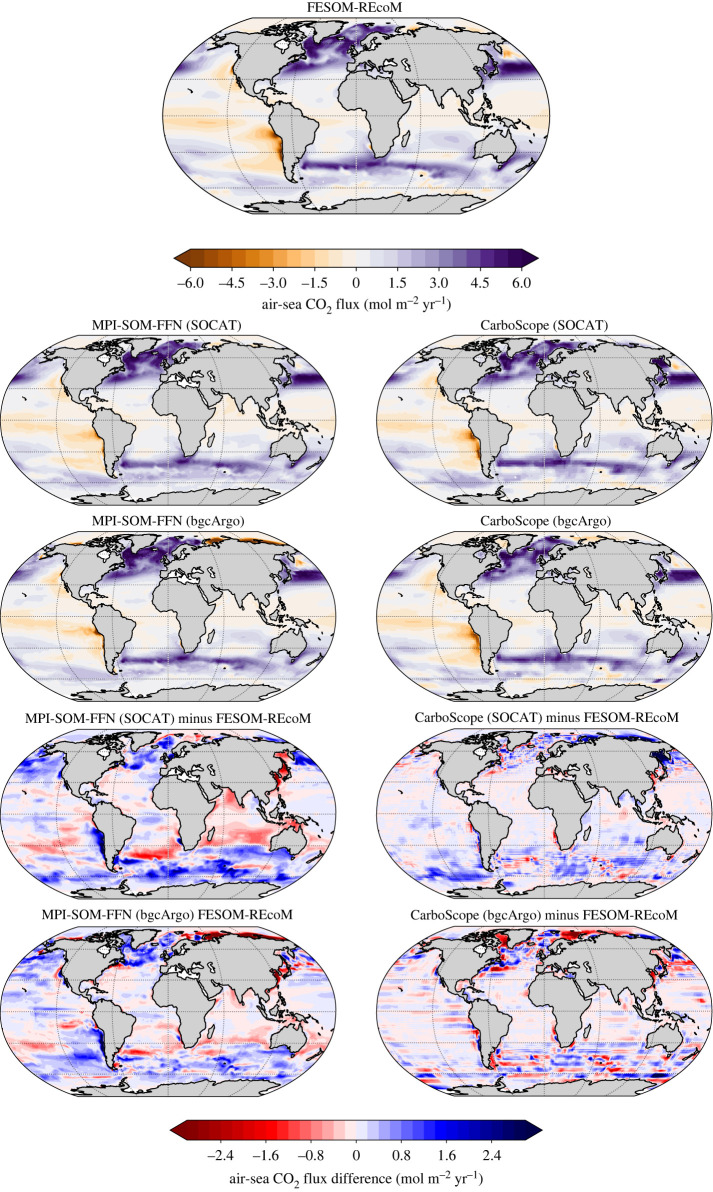
Air–sea ${\mathrm{CO}}_{2}$ flux in FESOM-REcoM and in the reconstructions by MPI-SOM-FFN (left) and CarboScope (right) products. Positive fluxes (purple) denote a flux into the ocean. In the difference maps, positive numbers (blue) denote a larger flux into the ocean (or a smaller flux out of the ocean) in the reconstruction than in FESOM-REcoM. Top: FESOM-REcoM, then from top to bottom: reconstructed air–sea ${\mathrm{CO}}_{2}$ flux in SOCAT sampling scheme, in bgcArgo scheme, and difference in air–sea ${\mathrm{CO}}_{2}$ flux between reconstruction and FESOM-REcoM in SOCAT and bgcArgo schemes, as indicated in the titles. Only the SOCAT and bgcArgo sampling schemes are shown. The differences between SOCAT and SOCAT+SOCCOM sampling schemes are small.

The spatial patterns of the air–sea ${\mathrm{CO}}_{2}$ fluxes averaged over the period 2009–2018 are very well reproduced by the MPI-SOM-FFN and CarboScope methods in all sampling schemes (figure 6). The only exception is anomalous outgassing in the Arctic in the bgcArgo scheme, which does not include any input data from this region. In MPI-SOM-FFN, the biases go generally into the same direction as the fluxes, i.e. fluxes into the ocean are overestimated in magnitude and fluxes out of the ocean are equally overestimated. Exceptions are a smaller outgassing signal along the west coast of South America and smaller uptake in the south Atlantic high uptake regions. A higher sampling density reduces the overestimation throughout the ocean, but reduces the overestimated outgassing more than the overestimated uptake. In CarboScope, biases are smaller but with the same patterns. However, the bias in the tropical outgassing regions is negligible, leaving the biases mostly located in high-wind ${\mathrm{CO}}_{2}$ uptake regions. In the bgcArgo scheme, patches of over- and underestimation alternate. We next inspect how well surface ${\mathrm{pCO}}_{2}$ is reconstructed, before investigating the role of transfer velocity in §3c.

### (b) Surface ${\mathrm{pCO}}_{2}$

**Figure 7.**
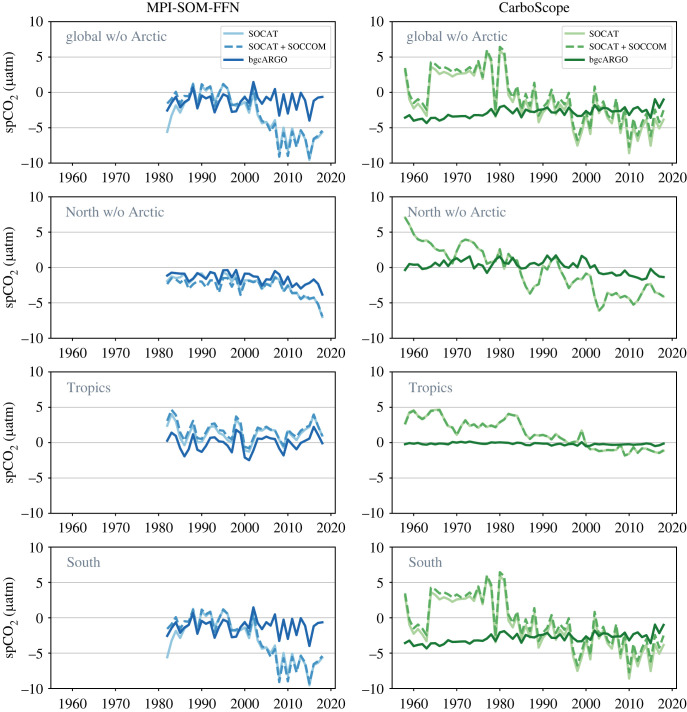
Biases in reconstructed annual mean surface ${\mathrm{pCO}}_{2}$ (μatm) (coloured lines) as calculated from the two ${\mathrm{pCO}}_{2}$ products minus the original FESOM-REcoM ${\mathrm{pCO}}_{2}$. The left column shows the MPI-SOM-FFN and the right column the CarboScope reconstructions for the three sampling schemes as indicated in the figures. From top to bottom: Global (excluding Arctic), North (excluding Arctic), Tropics, South.

**Figure 8.**
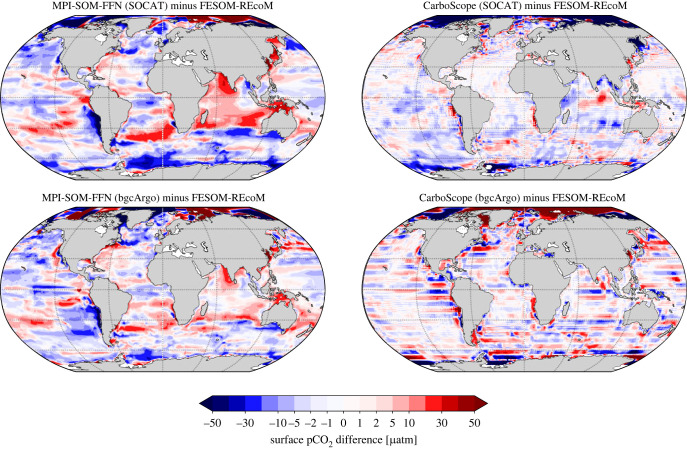
Spatial patterns of biases in reconstructed surface ${\mathrm{pCO}}_{2}$ (average 2009–2018) as calculated from the two ${\mathrm{pCO}}_{2}$ products minus the original FESOM-REcoM ${\mathrm{pCO}}_{2}$, for MPI-SOM-FFN (left) and CarboScope (right) in the sampling schemes SOCAT (top) and bgcArgo (bottom).

We inspect area-weighted ${\mathrm{pCO}}_{2}$ biases as the differences between regionally averaged (area-weighted) annual time series of the ${\mathrm{pCO}}_{2}$ reconstructions and the original FESOM-REcoM model output (figure 7), as well as spatial patterns of the 2009–2018 mean ${\mathrm{pCO}}_{2}$ bias (figure 8). We note that regionally averaged ${\mathrm{pCO}}_{2}$ biases are within −10 to +10 μatm outside the Arctic (figure 7). Locally, the biases can be larger (figure 8).

In all regions, the interannual variability of the bias remains unimproved from SOCAT to bgcArgo sampling distribution for MPI-SOM-FFN (figure 7). In CarboScope, the interannual varying component of the bias is reduced with improved data coverage. In the tropics, the ${\mathrm{pCO}}_{2}$ reconstruction is improved in the bgcArgo compared with the SOCAT sampling scheme in terms of the mean ${\mathrm{pCO}}_{2}$ (both products), and variability (CarboScope; figure 7). A larger positive ${\mathrm{pCO}}_{2}$ bias before 1985 and a small negative bias after 2000 in CarboScope (SOCAT) vanishes in the bgcArgo case. Thus, ${\mathrm{pCO}}_{2}$ is skilfully reproduced in the tropics with data availability as in the hypothetical bgcArgo sampling scheme.

In the North and the South, ${\mathrm{pCO}}_{2}$ is underestimated by both products in the regionally averaged time-series. In the North, the mean bias as well as a trend towards increasingly negative bias since 2005 is reduced in the bgcArgo relative to the SOCAT sampling scheme (both products). However, a bias in the mean of about −2 μatm persists in MPI-SOM-FFN and the bias varies between about −2 and +2 μatm in CarboScope. A similar result is found in the South, where the SOCAT (and SOCAT+SOCCOM) sampling scheme leads to an increasingly negative ${\mathrm{pCO}}_{2}$ bias between 2002 and 2008 in MPI-SOM-FFN (from near 0 to −8 μatm), which persists thereafter. This bias in the temporal evolution vanishes with more data availability in the bgcArgo case. The SOCCOM floats do not reduce this bias, which is located mostly in the Weddell Sea and coastal regions (figure 8). The Southern Ocean mean ${\mathrm{pCO}}_{2}$ bias is negative in the bgcArgo scheme, varying between −4 and +1 μatm in MPI-SOM-FFN and between −4 and −1 μatm in CarboScope (figure 7).

The maps of the mean ${\mathrm{pCO}}_{2}$ bias (figure 8) illustrate that the ${\mathrm{pCO}}_{2}$ biases are only partly co-located with the ${\mathrm{CO}}_{2}$ flux biases (figure 6). In fact, CarboScope ${\mathrm{pCO}}_{2}$ biases (outside of the Arctic) stem mostly from the Weddell and Ross Seas and a small region in the northwest Pacific. These Southern Ocean regions are usually ice-covered and it is unlikely that ${\mathrm{pCO}}_{2}$ biases in these regions lead to substantial biases in ${\mathrm{CO}}_{2}$ flux. In MPI-SOM-FFN, the negative ${\mathrm{pCO}}_{2}$ biases are more equally distributed in the Southern Ocean, but are also not co-located with the ${\mathrm{CO}}_{2}$ flux biases (figures 6 and 8).

In summary, we conclude from the analysis of ${\mathrm{CO}}_{2}$ flux and ${\mathrm{pCO}}_{2}$ biases that more and regularly spaced observations can cure or substantially reduce biases in the temporal evolution of the observation-derived ${\mathrm{CO}}_{2}$ flux estimates. However, even with regular $6^{\circ }\times 6^{\circ }$ sampling schemes, a discrepancy in the mean ${\mathrm{CO}}_{2}$ flux between FESOM-REcoM and MPI-SOM-FFN persists in the Southern Ocean, which suggests a methodological origin (figure 4). The ${\mathrm{pCO}}_{2}$ comparison (figures 7 and 8) suggests that the generally larger ${\mathrm{CO}}_{2}$ flux in the ${\mathrm{pCO}}_{2}$ products with near perfect sampling (bgcArgo) may be related to a persistent underestimation of ${\mathrm{pCO}}_{2}$, but with an additional imprint of high wind speed regions. The comparisons so far were conducted with the reconstructed ${\mathrm{pCO}}_{2}$ fields and a consistent use of the gas-exchange calculation as in FESOM-REcoM. Next, we investigate additional discrepancies in the mean ${\mathrm{CO}}_{2}$ flux, when using the method’s native gas-exchange calculations.

### (c) Gas-exchange calculation

**Figure 9.**
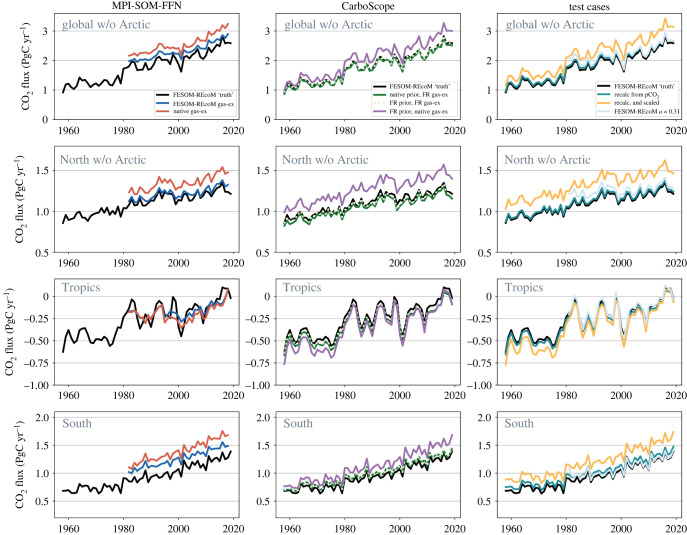
Effect of choices in gas-exchange calculations based on the ideal sampling case for (top to bottom:) global without Arctic, North without Arctic, Tropics, South. All panels show the ‘known truth’ of the FESOM-REcoM air–sea ${\mathrm{CO}}_{2}$ flux (flux integrated on native model mesh, black). Left column: MPI-SOM-FFN ${\mathrm{CO}}_{2}$ flux calculated from mapped ${\mathrm{pCO}}_{2}$ based on ideal sampling with either FESOM-REcoM piston velocity (blue) or with MPI-SOM-FFN native gas-exchange formulation (red). Middle column: CarboScope ${\mathrm{CO}}_{2}$ flux calculated from mapped ${\mathrm{pCO}}_{2}$ based on ideal sampling with its native prior from OCIM and FESOM-REcoM piston velocity (green, same as in figure 4), with FESOM-REcoM (FR) prior and FESOM-REcoM piston velocity (light grey, dashed), and with FESOM-REcoM prior and CarboScope native gas-exchange (purple). Right column: Test cases to quantify the error introduced by recalculating the air–sea ${\mathrm{CO}}_{2}$ flux from FESOM-REcoM ${\mathrm{pCO}}_{2}$ fields (full global coverage) and FESOM-REcoM piston velocity (green), from FESOM-REcoM ${\mathrm{pCO}}_{2}$ fields (full global coverage) and FESOM-REcoM gas-transfer velocity scaled to a global mean of $16.5\;{\mathrm{cm}\;h}^{-1}$ (orange). Also shown is a model experiment with higher gas-transfer coefficient a (0.31 instead of 0.251, light blue).

In addition to artefacts due to skewed data distribution, choices in gas-exchange calculations contribute to discrepancies between air–sea ${\mathrm{CO}}_{2}$ fluxes from mapping methods and from the ocean biogeochemistry model. The difference between air-sea ${\mathrm{CO}}_{2}$ flux calculated with FESOM-REcoM gas transfer velocity (as used for figures 4–6) and the MPI-SOM-FFN and CarboScope native gas-exchange calculation is not only substantial in magnitude, but also increases over time and affects the amplitude of interannual variations (figure 9). For example, the difference between the two gas-exchange calculations nearly doubles between 1982 and 2018 from $0.18\;{\mathrm{PgC}\;\mathrm{yr}}^{-1}$ to $0.34\;{\mathrm{PgC}\;\mathrm{yr}}^{-1}$ for the global flux in MPI-SOM-FFN, with roughly equal contributions from the North and South. The air–sea ${\mathrm{CO}}_{2}$ flux in the tropics is hardly affected. Similarly, the discrepancy between the two ways to calculate gas-exchange increases by more than a factor of five from $0.07\;{\mathrm{PgC}\;\mathrm{yr}}^{-1}$ to $0.42\;{\mathrm{PgC}\;\mathrm{yr}}^{-1}$ for the global flux in CarboScope. Here, the offset is larger in the North ($0.13\;{\mathrm{PgC}\;\mathrm{yr}}^{-1}$) than in the South ($0.06\;{\mathrm{PgC}\;\mathrm{yr}}^{-1}$) at the beginning of the time-series in 1958, but reaches similar levels towards the end of the time-series (around $0.22\;{\mathrm{PgC}\;\mathrm{yr}}^{-1}$). Note that the native gas-exchange experiment with CarboScope was conducted with a climatological FESOM prior rather than the OCIM prior, but the difference related to the choice of prior is small in the ideal sampling case (compare green and grey dotted lines in figure 9). Choice of prior affects mostly the period before 1990 in the SOCAT and SOCAT+SOCCOM sampling cases (see electronic supplementary material, figure S6). As a result, the amplitude of interannual variations is enlarged with the product’s native gas-exchange formulations, as is the trend since 2000 (figure 9, see also electronic supplementary material, figure S7 for the barplot equivalent to figure 5 but with MPI-SOM-FFN native gas-exchange calculation).

We further use the gridded FESOM-REcoM output to test the effects of offline gas-exchange calculation with monthly time stepping as is done in the MPI-SOM-FFN and most other products (CarboScope uses a daily time-step), and choice of the gas transfer coefficient (figure 9 third column). Differences between the direct model output (FESOM-REcoM, online calculation at every 15-min time-step) and the ${\mathrm{CO}}_{2}$ flux recalculated from monthly averaged ${\mathrm{pCO}}_{2}$ and gas-transfer velocity fields are on the order of 0.07 to $0.09\;{\mathrm{PgC}\;\mathrm{yr}}^{-1}$ in the Southern Ocean and even smaller elsewhere (compare green and black line in figure 9 third column). We interpret this difference as an averaging bias resulting from the high winds in the Southern Ocean whose effect is overestimated when the gas-exchange calculation is done on a monthly time-step. An interpolation bias in ${\mathrm{pCO}}_{2}$ and auxiliary fields may contribute as well (interpolation bias in ${\mathrm{CO}}_{2}$ flux is small, $0.03\;{\mathrm{PgC}\;\mathrm{yr}}^{-1}$). Tests with 6-hourly model output result in an estimate of the error associated with using monthly time-steps for gas-exchange calculation of 0.05–$0.06\;{\mathrm{PgC}\;\mathrm{yr}}^{-1}$ in the Southern Ocean and globally (as biases of $-0.03\;{\mathrm{PgC}\;\mathrm{yr}}^{-1}$ in the tropics and $+0.03\;{\mathrm{PgC}\;\mathrm{yr}}^{-1}$ in the North compensate). We note that this error is small, but it has the same spatial distribution as the ${\mathrm{CO}}_{2}$ flux bias (compare electronic supplementary material, figure S5 and figure 6) leading to ${\mathrm{CO}}_{2}$ uptake that is too strong in the temperate and high latitudes. We conclude that the procedure of using monthly fields to calculate gas-exchange works within reasonably smalluncertainty.

Another source of uncertainty is the recommended and commonly applied scaling of the coefficient of gas-transfer a to reach a global mean value of the gas-transfer velocity of $16.5\;{\mathrm{cm}\;h}^{-1}$ [53]. This scaling is applied in the native gas-exchange calculations in MPI-SOM-FFN and CarboScope. The coefficient of gas-transfer in FESOM-REcoM is not scaled and the gas-transfer velocity yields a long-term mean of $14.0\;{\mathrm{cm}\;h}^{-1}$. We test the effect of the scaling of the gas-exchange, which we approximate by scaling the gas-transfer velocity $k_{w}$ from FESOM-REcoM to $16.5\;{\mathrm{cm}\;h}^{-1}$ [53], rather than adjusting the coefficient of gas-transfer as commonly done [53,55]. This approach leads to a global flux increase by 0.17–$0.48\;{\mathrm{PgC}\;\mathrm{yr}}^{-1}$, and this difference also increases over time and stems in roughly equal amounts from the North and South (compare green and orange lines in figure 9 third column). A previous study [55] reported a difference in ${\mathrm{CO}}_{2}$ flux of $0.07\;{\mathrm{PgC}\;\mathrm{yr}}^{-1}$ between scaled and unscaled JRA forcing (note that this is a different version than the JRA55-do data set, which adjusts biases in JRA55 [9], used in FESOM-REcoM).

Further, we test the sensitivity of FESOM-REcoM to a higher value of the coefficient of gas-transfer a=0.31, which is taken as a value at the upper end of the previously reported range [52]. We perform a short test simulation 1981–2019, i.e. without a corresponding spin-up, and thus the results should be interpreted with some caution. Nevertheless, this test illustrates the low sensitivity of the ocean biogeochemistry model to choices in the coefficient of gas-transfer. The difference between the two simulations quickly decreases from about $0.25\;{\mathrm{PgC}\;\mathrm{yr}}^{-1}$ globally in the first years to reach $0.07\;{\mathrm{PgC}\;\mathrm{yr}}^{-1}$ at the end of the simulated period (compare black and light blue lines in figure 9 third column). The change over time is likely attributable to the lack of spin-up. This is a notably small effect in ${\mathrm{CO}}_{2}$ flux given that the global mean gas-transfer velocity is substantially higher with $17.2\;{\mathrm{cm}\;h}^{-1}$ relative to the $14.0\;{\mathrm{cm}\;h}^{-1}$ in the standard simulation. The reason for the low sensitivity of the ocean biogeochemistry model to the gas-transfer coefficient is that the ${\mathrm{pCO}}_{2}$ difference between ocean and atmosphere that determines the ${\mathrm{CO}}_{2}$ flux between these two compartments quickly adjusts to the initially higher flux [56]. The transfer of carbon between the surface mixed layer and the ocean interior is then the rate-determining step for air–sea ${\mathrm{CO}}_{2}$ flux in the model as in the real world. However, the regional distribution of the flux seems to be sensitive to the choice in gas-transfer coefficient, with the difference originating mainly from the North ($0.06\;{\mathrm{PgC}\;\mathrm{yr}}^{-1}$, figure 9).

All in all, the scaling of the gas-transfer coefficient to reach the best estimate of the global gas-transfer velocity explains the majority of the difference between the two products’ ${\mathrm{CO}}_{2}$ flux reconstructions with their native and FESOM-REcoM gas-transfer velocity. Additional uncertainty comes from the use of a different wind product in the native gas-exchange calculation in MPI-SOM-FFN (ERA5) than in FESOM-REcoM, and a different atmospheric ${\mathrm{pCO}}_{2}$ field in the native gas-exchange calculation of CarboScope. Using ERA5 may amount to a difference in the global mean flux of $0.13\;{\mathrm{PgC}\;\mathrm{yr}}^{-1}$ based on the assessment of Fay *et al.* [55].

## 4. Discussion

We investigated the uncertainty in ${\mathrm{pCO}}_{2}$ observation based estimates of the ocean carbon sink due to data sparsity. We show that two ${\mathrm{pCO}}_{2}$ mapping products using input data according to the SOCAT data distribution overestimate the global mean ${\mathrm{CO}}_{2}$ flux, and the trend since 2000 relative to the known model truth (figure 4). Our experiments suggest that the strong decadal variability, and particularly the reinvigoration of the Southern Ocean carbon sink [57] is to a large degree an artefact generated by sparse and decadally skewed data distributions, in line with the finding that the neural network based MPI-SOM-FFN method overestimates decadal variability in the Southern Ocean by 30% [31]. We relate the overestimation of the sink since the late 1990s to the skewed SOCAT data distribution towards low ${\mathrm{pCO}}_{2}$ (figure 3) that leads the neural network to also underestimate ${\mathrm{pCO}}_{2}$ by 5–10 μatm averaged over the Southern Ocean (figure 7). Interestingly, the dynamics of the reconstructed ocean carbon sink in the 'stagnation' period of the 1990s is less affected by data sparsity and appears to be real. The more robust reconstruction for the 1990s may be related to the distribution of observations in pCO_2_ space, which is not skewed towards low pCO_2_ values in the period 1982–1999 in our analysis (figure 3).

Even with the ideal sampling scheme, the mean Southern Ocean flux is consistently overestimated by about $0.2\;{\mathrm{PgC}\;\mathrm{yr}}^{-1}$ in MPI-SOM-FFN. This seems to be a methodological issue that may be related to the fact that MPI-SOM-FFN aims to minimize the global mismatch with SOCAT observations. While regional ${\mathrm{pCO}}_{2}$ biases average out in the first 20 years of the time-series (figure 7), the local ${\mathrm{pCO}}_{2}$ biases in high wind speed regions do not compensate in terms of ${\mathrm{CO}}_{2}$ flux (figure 6). Further, the biogeochemical provinces that merge regions from different parts of the globe with similar environmental conditions may contribute as well [23]. Southern Ocean mean flux and its trend are also reduced with the ideal sampling in CarboScope, but the effect is smaller than in MPI-SOM-FFN. This is because the CarboScope mixed layer scheme reproduces Southern Ocean ${\mathrm{pCO}}_{2}$ well even with the skewed data distribution. A mean ${\mathrm{pCO}}_{2}$ offset of −2 to −3 μatm in CarboScope (figure 7) stems from local biases in ice-covered regions which are less important for gas-exchange (figure 8). Similarly, in the North, which is the best observed area, a regularly spaced observation network and equal distribution in ${\mathrm{pCO}}_{2}$ space (figure 3) leads to reduction in ${\mathrm{pCO}}_{2}$ bias in both products (figure 7) and to a reduced ${\mathrm{CO}}_{2}$ flux trend since 2000 in MPI-SOM-FFN. The results illustrate that a process-based mapping method as CarboScope is less prone to biases due to data distribution. This is because a neural network can only reproduce patterns that are included in the training and target data set. With sparse and skewed observations, not all real world ${\mathrm{pCO}}_{2}$ values and dependencies on environmental variables are included in the training dataset. CarboScope normally uses a climatological alkalinity field, which is derived from sea surface salinity and temperature [50]. In our study, the model’s alkalinity is used as input to CarboScope, and hence additional uncertainty due to the relationship between alkalinity, salinity and temperature and due to missing interannual alkalinity variations in CarboScope are not included in our assessment. The use of data from SOCCOM floats (2014–2018) in addition to SOCAT has a negligible effect on the reconstructed ${\mathrm{pCO}}_{2}$ and ${\mathrm{CO}}_{2}$ flux (figures 4 and 5). However, an idealized sampling scheme with 1000 regularly ($6^{\circ }\times 6^{\circ }$) spaced sampling sites [36] with high-accuracy ${\mathrm{pCO}}_{2}$ measurements would be sufficient to reduce the sampling and methodological bias of the mean ${\mathrm{CO}}_{2}$ flux to 2–9% globally, and to 5–17% in the Southern Ocean. This would also lead to a reasonably good estimate of the amplitude and particularly the phasing of ${\mathrm{CO}}_{2}$ flux variability (figure 5).

Despite the good reconstruction of surface ${\mathrm{pCO}}_{2}$ in the ideal (‘bgcArgo’) sampling scheme, a mean and growing offset in ${\mathrm{CO}}_{2}$ flux of 0.4 to $0.6\;{\mathrm{PgC}\;\mathrm{yr}}^{-1}$ at the end of the time-series remains between the ‘known-truth’ and the products when using the products native gas-transfer velocity. For MPI-SOM-FFN, this offset can be attributed to methodological biases and gas-exchange uncertainty in about equal amounts. For CarboScope, the discrepancy is entirely due to choices in gas-exchange calculation. All contributions work towards a discrepancy particularly in the high latitudes. This is in agreement with reported uncertainties of the ${\mathrm{pCO}}_{2}$ observation-based estimates of $0.45\;{\mathrm{PgC}\;\mathrm{yr}}^{-1}$–$0.6\;{\mathrm{PgC}\;\mathrm{yr}}^{-1}$ with a dominant contribution from the gas-transfer velocity [48,53,55,58].

The 1-σ uncertainty of ${\mathrm{pCO}}_{2}$-based ${\mathrm{CO}}_{2}$ flux estimates in the Global Carbon Budget [2] is quantified as a contribution of random uncertainty ($0.3\;{\mathrm{PgC}\;\mathrm{yr}}^{-1}$, standard deviation of ${\mathrm{pCO}}_{2}$ product ensemble) uncertainty in ${\mathrm{pCO}}_{2}$ measurements ($0.2\;{\mathrm{PgC}\;\mathrm{yr}}^{-1}$, [59]), gas-transfer velocity k ($0.2\;{\mathrm{PgC}\;\mathrm{yr}}^{-1}$, [59], wind product ($0.1\;{\mathrm{PgC}\;\mathrm{yr}}^{-1}$, [55]), uncertainty in river flux adjustment ($0.3\;{\mathrm{PgC}\;\mathrm{yr}}^{-1}$, 2-σ [5]) and ${\mathrm{pCO}}_{2}$ mapping ($0.2\;{\mathrm{PgC}\;\mathrm{yr}}^{-1}$, [23]), resulting in a total 1-σ uncertainty of $0.6\;{\mathrm{PgC}\;\mathrm{yr}}^{-1}$. Our analysis illustrates that the total uncertainty is increasing over time and may be $0.6\;{\mathrm{PgC}\;\mathrm{yr}}^{-1}$ even when the river flux adjustment is not needed as in our study design. Based on the subsampling experiments conducted here, the mapping uncertainty may be an underestimate and an additional uncertainty term due to sparse and skewed data distribution of $0.2\;{\mathrm{PgC}\;\mathrm{yr}}^{-1}$ (difference between mean flux in SOCAT and ideal sampling schemes) should be added to this assessment (§3a, figure 5).

The discrepancy between the ocean carbon sink estimates by the ten global ocean biogeochemistry models (such as FESOM-REcoM) and the 8 surface ${\mathrm{pCO}}_{2}$-based data products (such as MPI-SOM-FFN and CarboScope) in the Global Carbon Budget is large [2]. While they matched reasonably well in the 1990s, they have diverged after 2000. The full range of all estimates amounts to $2\;{\mathrm{PgC}\;\mathrm{yr}}^{-1}$ in 2021, and the difference between the GOBM and pCO${\;}_{2}$-product ensemble means to $1\;{\mathrm{PgC}\;\mathrm{yr}}^{-1}$. It is estimated that the GOBMs underestimate the ocean carbon sink by 10–20% as evidenced by a direct comparison to interior ocean anthropogenic carbon accumulation [2,3]. This is also supported by higher estimates from atmospheric inversions (although they often use ${\mathrm{pCO}}_{2}$ products as priors, and are thus equally affected by the uncertainties studied here) and $O_{2}:N_{2}$ ratios [2,4]. Analysis of Earth System Models (which may differ from the biases in GOBMs forced by atmospheric reanalysis) indicate that this underestimation may be explained by biases in ocean carbon transport and mixing [11–13], biases in the chemical buffer capacity [15], and by a late starting date of the simulations [60]. Here, we demonstrate that the divergence since the early 2000s can to a large degree be explained by the overestimation of the trend by the ${\mathrm{pCO}}_{2}$ products. This effect is stronger in the neural network method than in CarboScope. The effect of sparse and unevenly distributed observations on other neural network and cluster regression methods that contribute to the Global Carbon Budget [24,28,61] remains to be tested. The MPI-SOM-FFN product shows by far the largest ${\mathrm{CO}}_{2}$ flux trend since 2000 compared with the other GCB2022 ${\mathrm{pCO}}_{2}$ products (table 1), although the exact numbers depend on chosen start date. However, cluster-regression (OS-ETHZ-GRaCER) and neural network (CMEMS-LESCE-FFNN) approaches can also have trend estimates on the lower end of the spectrum (table 1), reflecting the role of methodological choices within these methods. Yet, all ${\mathrm{pCO}}_{2}$ products show a larger trend than the GOBM ensemble mean and also higher than the GOBM with the largest trend (table 1). Notably, the standard deviation of the $pCO_{2}$ products’ trends is an order of magnitude larger than of the GOBMs, illustrating that the trend is poorly constrained by the ${\mathrm{pCO}}_{2}$ products. The trend in the GOBM FESOM-1.4-REcoM ($0.43\;{\mathrm{PgC}\;\mathrm{yr}}^{-1}\;{\mathrm{decade}}^{-1}$) that is used here, falls close to the GOBM ensemble average ($0.41\;{\mathrm{PgC}\;\mathrm{yr}}^{-1}\;{\mathrm{decade}}^{-1}$) and close to the successor version FESOM-2.1-REcoM2 ($0.45\;{\mathrm{PgC}\;\mathrm{yr}}^{-1}\;{\mathrm{decade}}^{-1}$). This indicates that the data distribution plays a role in the full set of observational products within the Global Carbon Budget. A ${\mathrm{pCO}}_{2}$-product based on ${\mathrm{pCO}}_{2}$ mismatches between GOBMs and observations [62] clusters with a few products estimating a somewhat slower growing ocean carbon sink, but still close to the product ensemble average and well above any GOBM estimate (table 1). In conclusion, we advocate for continued ${\mathrm{pCO}}_{2}$ mapping intercomparisons [29] and observation-system design experiments [31,32] to understand the biases in all mapping methods. The ensemble of global ocean biogeochemistry models forced with atmospheric reanalysis and observed atmospheric CO${\;}_{2}$ as used in the GCB are closer to interannual climate and carbon variability than Earth System Models, and thus are an ideal tool for such experiments. While the results on data distribution may be dependent on the simulated ${\mathrm{pCO}}_{2}$ in the ocean model used, the subsampling experiment demonstrates the sensitivity of the mapping products to sparse and unevenly distributed observations, as well as uncertainties in gas-exchange choices.

In terms of decadal and interannual variability, the ${\mathrm{pCO}}_{2}$ products are skilful when driven with sufficient and evenly distributed ${\mathrm{pCO}}_{2}$ observations. We note that the metric for amplitude of variability (standard deviation of detrended time-series) does not separate between interannual and decadal variability and thus needs to be interpreted with some caution. Inspection of the time-series (figure 4) indicates the reduction of erroneous decadal variability and slight improvement in interannual variability in the ideal bgcArgo sampling scheme (except for MPI-SOM-FFN in the tropics). We further note that also the estimate of variability and trend is affected by choices in gas-exchange calculations.

In agreement with previous studies of observation system design [31–33], we emphasize the need for a sustained and coherent network of high-quality ${\mathrm{pCO}}_{2}$ observations. Ship-based measurements provide high-accuracy data, but are too sparse in many regions of the ocean, especially in the Southern Hemisphere. A combination of multiple platforms will be needed including autonomous devices, such as biogeochemical Argo floats, but also saildrones [37], wave gliders, moorings. We demonstrate that an observation network with a coverage similar to the planned global deployment of 1000 biogeochemical Argo floats may be sufficient to fill the gaps in the current ship-based networks. In reality, the network will not follow such a regular distribution, and high accuracy ${\mathrm{pCO}}_{2}$ measurements will be needed to at least partially fill the gaps in the Southern Ocean. The bgcArgo floats are equipped with pH sensors, and hence ${\mathrm{pCO}}_{2}$ needs to be derived from pH and a multi-linear regression-derived alkalinity estimate. Currently, this procedure is associated with uncertainties that are substantially larger than for the ${\mathrm{pCO}}_{2}$ measurements on ships [18,35,63]. Our analysis reveals that systematic biases of around 5 μatm can lead to large errors in reconstructed ${\mathrm{pCO}}_{2}$ and ${\mathrm{CO}}_{2}$ flux. As the array of biogeochemical Argo floats expands, it will be essential to conduct on-going intercomparisons between float and ship-based measurements with the goal of developing an unbiased multi-platform sampling array that captures the relevant spatial and temporal variability. This may involve more extensive sampling of surface pH and alkalinity on research vessels and ships of opportunity to allow better assessment of float pH and salinity-derived alkalinity and targeting float locations for purposeful crossover comparisons.

## 5. Conclusion

We have assessed the sensitivity of surface ocean ${\mathrm{pCO}}_{2}$ reconstructions to sparse observations and uneven sampling in space and time. The Southern Ocean decadal variability stands out as being especially prone to such uncertainties, and this is stronger in the neural network compared with the process-based mapping scheme. We thus caution the reader to not relate to ${\mathrm{pCO}}_{2}$ products as observations, but to acknowledge that they are statistical models of sparse observations. There are two possible ways forward that both need to be addressed in order to improve robustness of the ocean carbon monitoring system. First, the observational network must be strengthened, with sustained and operational funding and with a combination of multiple platforms. Notably, the total number of global high-quality ocean ${\mathrm{CO}}_{2}$ observations has surpassed the number of observations in our tested ‘ideal’ sampling design, indicating that the considerate distribution of sampling sites is more important than simply adding more observations in the same places. Secondly, the mapping methods should be routinely evaluated for their ability to handle skewed data distribution, and should be improved when necessary.

## Data Availability

The underlying data sets (sampling masks, FESOM-REcoM output fields, MPI-SOM-FFN and CarboScope pCO_2_ and air–sea CO_2_ flux reconstructions for the three sampling masks) are deposited at doi:10.5281/zenodo.7784745 [64]. Additional figures are provided in electronic supplementary material [65].
